# Multiomic analysis of HER2-enriched and AR-positive breast carcinoma with apocrine differentiation and an oligometastatic course: a case report

**DOI:** 10.3389/fonc.2023.1240865

**Published:** 2023-07-31

**Authors:** Brando Poggiali, Agostino Ponzetti, Marica Malerba, Fabio Landuzzi, Federica Furia, Debora Charrance, Sara Trova, Vittoria Perseghin, Patrizia A. Falcone, Valentina Alliod, Alessandra Malossi, Pierpaolo Carassai, Ubaldo Familiari, Manuela Vecchi, Stefano Gustincich, Marina Schena, Andrea Cavalli, Alessandro Coppe

**Affiliations:** ^1^ CComputational and Chemical Biology, Italian Institute of Technology (IIT), CMP^3^VdA, Aosta, Italy; ^2^ Oncologia e Ematologia oncologica, Ospedale Umberto Parini, Aosta, Italy; ^3^ Non-Coding RNAs and RNA-Based Therapeutics, Italian Institute of Technology (IIT), CMP^3^VdA, Aosta, Italy; ^4^ Biologia Molecolare Oncologica e Virologica, Analisi Cliniche, Ospedale Umberto Parini, Aosta, Italy; ^5^ Anatomia Patologica, Ospedale Umberto Parini, Aosta, Italy; ^6^ Centre Européen de Calcul Atomique et Moléculaire (CECAM), Ecole Polytechnique Fé dérale de Lausanne, Lausanne, Switzerland

**Keywords:** breast cancer, personalized medicine, NGS, bioinformatic analysis, apocrine carcinoma, case report, WGS, nanopore

## Abstract

Breast carcinoma is the most prevalent cancer among women globally. It has variable clinical courses depending on the stage and clinical-biological features. This case report describes a 56-year-old female with invasive breast cancer without estrogen or progesterone receptor expression, with apocrine differentiation, and with no germline variants in the BRCA1 and BRCA2 genes. Throughout the clinical course, the patient exhibited discordant results for HER2 in immunohistochemistry and *in situ* hybridization. During the second relapse, the disease displayed apocrine microscopic features. The tumor underwent analysis for the androgen receptor, GCDFP-15, RNA-seq, and whole-genome sequencing (WGS) to identify the breast cancer subtype and to characterize the cancer genome. Our bioinformatic analysis revealed 20,323 somatic SNV/Indels, including five mutations in cancer-related genes that are believed to be responsible for the tumor’s development. Two of these mutations were found in the *PIK3CA* and *TP53* genes. Furthermore, the tumor tissue exhibited large copy number alterations to the chromosomes, which could impact gene expression through complex mechanisms and contribute to the tumor phenotype. Clustering algorithms applied on RNA-sequencing data categorized this cancer as a HER2+ subtype. The second-line capecitabine chemotherapy treatment is ongoing, and the patient is responding well. Bioinformatic results support the current treatment decision and open the way to further treatments.

## Introduction

Breast cancer is the most common cancer in women, with over 2.2 million new cases and 684,996 deaths reported in 2020 (1). While early-stage (non-metastatic) breast cancer is considered curable, advanced or metastatic breast cancer remains incurable, with a 5-year survival rate of only 38% (2). However, therapeutic strategies are available. Their main goal is prolonging survival and maintaining quality of life, and even better results can be obtained in the oligometastatic setting (3, 4).

The tumor’s histological and molecular characteristics influence breast cancer treatment decisions. Indeed, breast cancer is a molecularly heterogeneous disease, and several classifications have been developed to group tumors based on their molecular features. Sørlie et al. classified breast cancers based on their gene expression profile (5) into five intrinsic subtypes: Luminal A, Luminal B, HER2-enriched, Basal-like, and Normal breast-like. Each category has well-defined classical immunochemistry markers, such as estrogen receptor (ER), progesterone receptor (PR), human epidermal growth factor receptor 2 (HER2), and Ki-67 (6). Invasive apocrine carcinoma is a rare subtype of non-luminal breast cancer. Invasive apocrine carcinoma is HER2-positive in ~30% of cases and displays significant biological aggressiveness, potentially related to the activation of the androgen receptor (AR) pathway (7, 8). Identifying the correct molecular subtype is crucial for treatment decisions because each subtype has specific therapeutic targets and different prognoses (9). Furthermore, germline mutation in *BRCA1/2* and somatic mutations, such as copy number alterations (CNAs) and single nucleotide variants (SNV) in driver genes (e.g. *TP53*, *PIK3CA*), have prognostic relevance for the therapy outcome and survival (10–13), including in subtypes like apocrine carcinoma (14). The complexity of breast cancer and its variability in responding to different treatments underscores the need for personalized medicine.

In this paper, we present a case report of a patient with an ER/PR-negative invasive breast cancer, which, from a clinical-molecular perspective, exhibited a discordant HER2 status and expression of the AR. At the second relapse, the tumor displayed partial apocrine features and a pathogenetic *PIK3CA* mutation. The clinical course could be defined as oligometastatic, and the response was obtained with both first-line paclitaxel and second-line capecitabine combined with radiotherapy.

At the second relapse, we performed both whole-genome and transcriptome sequencing analyses to fully characterize the tumor’s genomic variations and gene expression. Our findings provide insight into the molecular characteristics of this unique breast cancer subtype and may contribute to the development of more effective personalized treatment strategies.

## Case presentation

**Figure 1:**
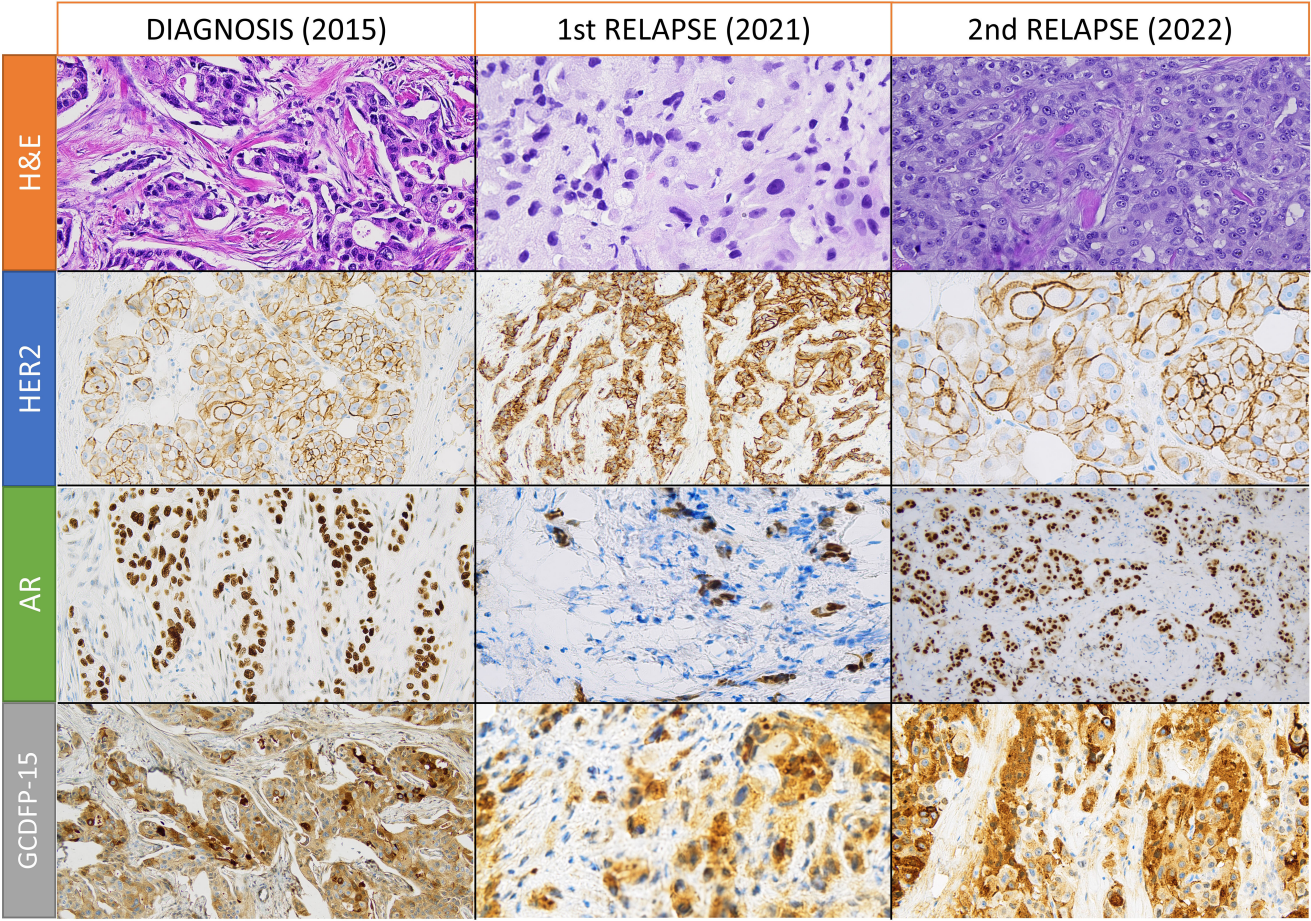
Hematoxylin and eosin (H&E) and immunohistochemical stainings for human epidermal growth factor receptor 2 (HER2), androgen receptor (AR), and gross cystic disease fluid protein 15 (GCDFP-15) in samples from diagnosis, first relapse, and second relapse. All images are obtained with 200X magnification, except for HER2 and AR stainings of the second relapse (obtained with 400X and 100X magnification, respectively).

We present the case of a 56-year-old female with no significant medical history or family history of cancer. During routine screening in November 2015, she was diagnosed with localized left breast cancer. The patient underwent a breast-wide excision with sentinel lymph node biopsy, revealing a breast carcinoma not otherwise specified (NOS), grade 2 according to Elston-Ellis classification, without expression of ER or PR, and with a HER2 score of 2+ (**Figure 1**) but without amplification at fluorescence *in situ* hybridization (FISH). The Ki-67 labeling index was 40%, and the disease was classified as pT1c-pN1(sn). Post-operative computed tomography scan and bone scintigraphy showed no signs of distant metastasis. From February to August 2016, the patient received adjuvant chemo-radiotherapy with epirubicin, cyclophosphamide, and paclitaxel. This was followed by 45 Gy in 20 fractions on the left breast plus 5 Gy boost on the surgical bed. *BRCA1* and *BRCA2* analysis showed no mutations.

During follow-up, in December 2020, a left supraclavicular lymph node appeared and an 18-fluorodeoxyglucose (18-FDG) positron emission tomography-computed tomography (PET-CT) scan confirmed the disease relapse in five non-bulky supraclavicular and retropectoral lymph nodes. A lymph-nodal ultrasound-guided core biopsy revealed malignant cells from breast carcinoma that were ER-negative and PR-negative with a HER2-score of 3+ (**Figure 1**) but without gene amplification at chromogenic *in situ* hybridization (CISH). PD-L1 staining with Ventana SP142 clone was negative on tumor-infiltrating lymphocytes with an IC score of less than 1%.

After discussing the risks and benefits of a single-agent chemotherapy with the patient and her relatives, she received first-line chemotherapy with Paclitaxel from February 2021 to January 2022. This produced a partial response to the disease; however, the chemotherapy caused persistent maximum grade (maxG) 2 peripheral neuropathy.

In February 2022, ultrasonography and PET-CT confirmed the clinical suspicion of oligoprogression in a new supraclavicular lymph node at a site anterior to the previous site, and in two internal mammary nodes.

After a multidisciplinary discussion, supraclavicular lymph node surgical excision was performed, and histopathological analysis showed a gross cystic disease fluid protein 15 (GCDFP-15) positive breast carcinoma with partial apocrine differentiation and expression of the AR in 95% of cells. The carcinoma was ER-negative, PR-negative, and HER2 3+, but without amplification at CISH and FISH. A revision was performed in a referral center in Turin, Italy, and the HER2 IIC was downstaged to 2+ disease. The histopathological samples from breast disease (2015) and the first lymph nodal relapse were re-assessed and showed positivity for the AR and GCDFP-15.

Next-generation sequencing (NGS) analysis was performed on the metastatic supraclavicular lymph node using *Myriapod NGS Cancer Panel DNA*. This showed a *PIK3CA* p.His1047Arg mutation with an allelic frequency of 26.39%. After excluding a dihydropyrimidine dehydrogenase (DPYD) polymorphism, the patient began receiving second-line capecitabine in April 2022. From June to July 2022, consolidation radiotherapy was performed on the left infra-supraclavicular region and a local boost of 9 Gy with maxG2 fatigue, maxG1 hand-foot syndrome grade, and maxG2 radiodermatitis.

In November 2022, after the sixth cycle of capecitabine, a PET-CT scan with 18-FDG showed a metabolic response at all disease sites, without any pathological FDG-capitation.

The second-line chemotherapy is ongoing.

## Genomic profiling of the tumor

Genomic DNA was extracted from peripheral blood (control) and the metastatic supraclavicular lymph node, obtained from the second relapse. Whole-genome sequencing (WGS) was performed on the two samples using a PCR-free library approach and the Novaseq 6000 System with target coverages of 60X and 120X for the blood and tumor samples, respectively. The NVIDIA Clara Parabricks pipelines were used to identify germline and somatic variants, which were annotated and filtered using an in-house pipeline (see **Supplementary Materials**).

No pathogenic or likely pathogenic germline variants were found in breast cancer predisposition genes including *BRCA1* and *BRCA2* (15). After subtracting the germline variants from the metastatic tumor sample, we detected 20,323 somatic mutations (16,544 SNVs and 3,779 Indels). In particular, we identified five mutations in five cancer-related genes (see **Table 1**) that could be responsible for the tumor’s development. Two of these mutations were classified as pathogenic according to CLINVAR (16): a *PIK3CA* mutation with a variant allele frequency of 34.17%, and a *TP53* mutation with a variant allele frequency of 55.71%. CLINVAR did not classify the mutations in *NFB2*, *ATM*, or *BTK* as clinically significant. CANCERVAR software classified the mutation in *NFKB2* as having uncertain significance (tier III) based on three types of evidence. It classified the mutation in *ATM* as potentially clinically significant (tier II) based on 8 types of evidence. Lastly, the mutation in *BTK* was classified as having uncertain significance based on seven types of evidence.

**Table 1:** Somatic variants obtained from the WGS bioinformatic analysis.

GENE	CHR	POSITION	REF	ALT	HGVS.c	HGVS.p	COVERAGE	ALLELE COVERAGE	FREQUENCY	CLINVAR	CLINVAR STATUS	CANCERVAR	gnomAD	CADD
PIK3CA	chr3	179234297	A	G	c.3140A>G	p.His1047Arg	120	79,41	34.17 %	Pathogenic	3	Tier_II_potential [10]	.	22.5
TP53	chr17	7675088	C	T	c.524G>A	p.Arg175His	70	31,39	55.71 %	Pathogenic	2	Tier_I_strong [11]	1.548e-05	23.4
NFKB2	chr10	102400117	G	CLI	c.1507G>C	p.Val503Leu	68	35,33	48.53 %	.	-1	Tier_III_Uncertain [3]	.	23.7
ATM	chr11	108312478	G	C	c.5986G>C	p.Glu1996Gln	106	58,48	45.28 %	.	-1	Tier_II_potential [8]	.	24.1

**Figure 2:**
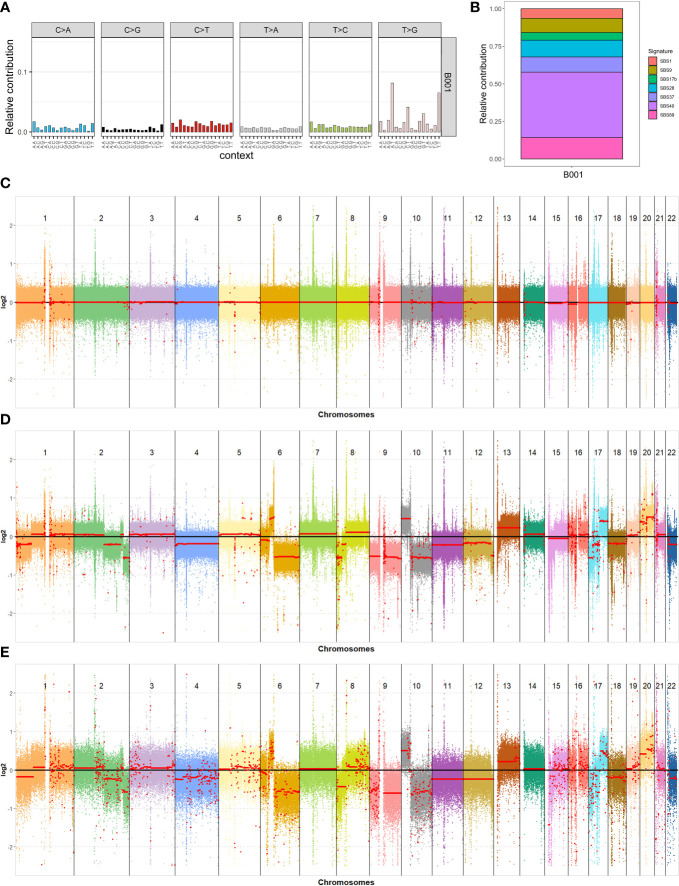
Genomic analysis of the blood and tumor samples. **(A)** 96-mutational profile; **(B)** Relative contribution of the different mutational signatures identified after the decomposition of the 96-mutational profile; **(C–E)** Results of the copy number variations analysis for all autosomal chromosomes: blood and tumor samples using short reads data **(C, D)** and tumor sample using long reads data **(E)**, respectively.

To investigate the type of somatic mutations and the processes that generated them, we performed a mutational signatures analysis using R (www.r-project.org) and the MutationalPatterns (17) R/Bioconductor package. We analyzed 16,544 SNVs for mutational changes and sequence context, and generated a plot that represents the abundance of somatic SNVs in trinucleotide contexts, which is also defined as a 96-mutational profile (see **Figure 2A**). The most abundant nucleotide change was T>G, and the most enriched mutational trinucleotide contexts were ATT and TTT sequences. We decomposed the 96-mutational profile into different mutational signatures stored in COSMIC (18). Their relative contributions are shown in **Figure 2B**. We detected seven substitution mutational signatures (SBSs) in the tumor sample: SBS1, SBS9, SBS17b, SBS28, SBS37, SBS40, and SBS89. SBS40 contributed the most to the 96-mutational profile. The number of mutations attributed to SBS40 correlates with patient age in different types of human cancer (18), although the etiology is unknown. SBS1 is attributed to the deamination of 5-methylcytosine and is a clock-like signature, with the number of mutations correlating with age in normal cells and cancer cells (19). SBS9 seems to be due to the activity of polymerase η (18). The etiology of SBS17b, SBS28, SBS89, and SBS37 is unknown. However, SBS17b is associated with fluorouracil (5FU) chemotherapy treatment and damage inflicted by reactive oxygen species (ROS) (20).

We used CNVkit software (21) to analyze copy number variations (CNVs) in the genome. The copy number profiles for all autosomal chromosomes in the blood and tumor samples are shown in **Figures 2C, D**, respectively. Each colored dot represents the *log2* ratio in a sequence range of 750 bp, while the red line is the average *log2* ratio on a broader region. The red line of blood chromosomes lies exactly on the *log2* ratio value of zero, which means that they do not have any CNAs. In contrast, the tumor shows large CNVs in the chromosomes. Our focus was on cancer-associated genes located in chromosomal regions detected with CNV analysis. We identified 2,414 genes in duplicated regions, with 1294 of these coding for proteins, and 1528 genes in deleted regions, with 768 of these coding for proteins (**Table S1**). To identify potential tumor-specific genes, we filtered these genes using a list of 723 cancer-associated genes from the Cancer Gene Census website (https://cancer.sanger.ac.uk/census), resulting in 45 duplicated and 90 deleted genes (**Table S2**). We decided to perform CNV analysis in the tumor sample using data from third-generation sequencing technology. This was to reduce problems related to the short-read sequencing methodology in the CNV analyses, such as secondary alignments due to highly repetitive regions and technical duplicates. The tumor sample was sequenced, and we obtained a genome coverage of 27.54X, an N50 length of 45,147 bp, and a mean length of 17,518.9 bp. The CNV analysis was performed by setting a bin of 10,000 bp to reduce the high variance of the *log2* ratio in each bin probability due to the lack of a reference and the lower coverage relative to the Illumina experiment. Nevertheless, the CNV analysis obtained with PromethION 24 (Oxford Nanopore Technologies) showed large chromosome alterations to the tumor genome (**Figure 2E**), which strongly correspond to the alteration detected using Illumina sequencing data (**Figure 2D**).

Finally, tumor mutational burden (4.65 muts/Mb) and microsatellite instability (0.04% of mutated microsatellites) analyses did not show any relevant results (see **Supplementary Materials**).

## Gene expression analysis of the tumor sample

To gain insight into the gene expression profile of the metastatic tumor sample, we conducted a comprehensive analysis using RNA sequencing (RNA-Seq) with the NovaSeq 6000 System (Illumina) and an in-house pipeline (**Supplementary Materials**). To supplement our analysis, we incorporated publicly available datasets, including a total of 28 healthy breast tissue samples (including 15 adjacent noncancerous tissues) downloaded from NCBI SRA (project accession numbers: PRJNA292118, PRJNA855324, PRJNA839244). We also used RNA-sequencing data from 1085 breast cancer patients obtained from the TCGA datasets (see **Supplementary Materials**).

Next, we compared the expression levels of the tumor-specific genes located in CNVs in our tumor sample to those of 10 healthy breast tissue samples from the PRJNA855324 and PRJNA839244 projects. In the set of 47 duplicated genes, we detected 24 genes in our tumor sample with higher expression levels than healthy breast tissues (**Table S3**), while 2 genes had lower expression levels. For the deleted genes, 25 had a lower expression, and 4 had a higher expression (**Table S4**).

**Figure 3:**
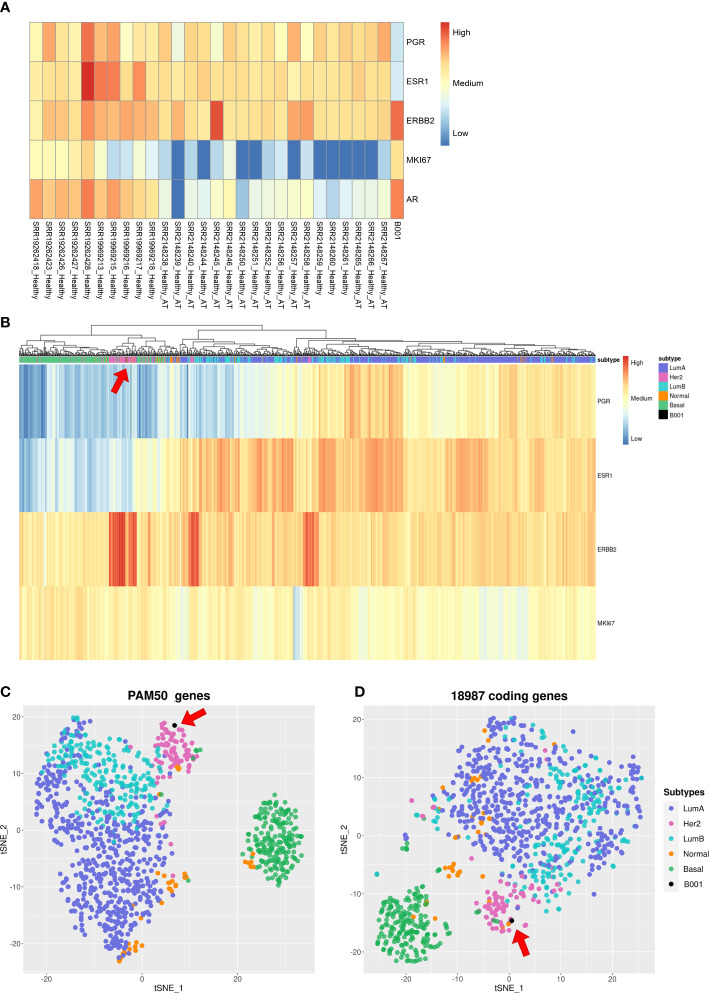
Gene expression analysis of the tumor sample. **(A)** Hierarchical clustering heatmap with gene expression levels of *PGR*, *ESR1*, *HER2*, *MKI67*, and *AR* in breast cancer tissue from 28 healthy patients and from our tumor sample; **(B)** Hierarchical clustering heatmap showing gene expression levels of *PGR*, *ESR1*, *HER2*, and *MKI67* of 1085 breast cancer patients in the TCGA dataset and our tumor sample; **(C, D)** T-SNE algorithm with 1085 breast cancer patients in the TCGA dataset and our tumor sample using PAM50 genes and 18987 coding genes, respectively.

We also examined the expression levels in the tumor sample of critical genes involved in breast cancer, including *Progesterone Receptor* (*PGR*), *Estrogen Receptor 1* (*ESR1*), *Human Epidermal growth factor Receptor 2* (*HER2*, also known as *ERBB2*), *Marker Of Proliferation Ki-67* (*MKI67*), and *AR*. Subsequently, we compared these levels with those observed in 28 samples of healthy breast tissue. The resulting heatmap (**Figure 3A**) showed that the tumor sample had low expression levels for *PGR* and *ESR1*, medium expression levels for *MKI67*, and high expression levels for *HER2* and *AR*.

To further understand the molecular subtype of our tumor sample, we performed cluster analysis of 1085 breast cancer patients from TCGA datasets using different sets of genes. **Figure 3B** reports a heatmap built with the four genes used to classify the breast cancer subtypes: *PGR*, *ESR1*, *HER2*, and *MKI67*. These four genes capture a structure in the data: there is a light green cluster containing Basal-like patients, a pink cluster with HER2+ patients, and on the right branch of the dendrogram there are Luminal A and Luminal B samples, which are not separated. Our sample is in the pink cluster close to the HER2+ patients. **Figure 3C** shows a t-distributed stochastic neighbor embedding (t-SNE) plot built using PAM50 genes. In this case, we see: i) a well-defined cluster related to Basal-like and HER2+ patients; ii) Luminal A and Luminal B clusters that partially overlap; and iii) Normal-like patients with no well-defined location in the plot. Our sample is in the HER2-enriched cluster. To gain a complete picture of the tumor’s gene expression profile, we analyzed 18,987 coding genes in the human genome using the t-SNE statistical method. While the resulting clusters were not well-defined for all subtypes, we were still able to identify a cluster of patients with Basal-like subtypes and a blurry cluster of HER2+ individuals, which contained our sample (**Figure 3D**).

## Discussion

Adapting the clinical and molecular classification of breast cancer to a single patient’s disease is one of the most difficult tasks for medical and molecular oncologists.

In this study, we investigated the deep molecular issues of a patient with a metastatic ER-negative and PR-negative breast cancer with partial apocrine differentiation, in agreement with GCDFP-15 expression, and an oligometastatic clinical course. Since the diagnosis, the disease exhibited a moderate (2+) or strong (3+) expression of the HER2 protein, but CISH or FISH analyses were negative, thus indicating a HER2-low disease (22). After more than 20 years of clinical use of anti-HER2, emerging data and improved analytical methods mean that the dichotomous classification of a positive or negative HER2 clinical category is evolving towards a continuum (23, 24). Here, our patient did not receive any anti-HER2 antibodies associated with conventional chemotherapy. At diagnosis, this choice was based on the HER2 2+/FISH-negative result. At relapse, despite the HER2 3+ result, CISH was performed (FISH was not available at our center at that time) because of the slow and oligometastatic behavior. Single-agent paclitaxel was then administered with a progression-free interval of nearly one year. At the second oligometastatic relapse, the case was revised in a referral center. Both CISH and FISH for *HER2* were performed, again with negative results. Thus, systemic treatment with single-agent capecitabine was chosen because of the possibility of concurrent radiotherapy, obtaining a complete metabolic response.

The tumor genome revealed a complex picture, with 20,323 somatic mutations (16,544 SNVs and 3,779 Indels). We found an enrichment of T>G nucleotide change in the SNVs, and mutational signature analysis associated some of the somatic mutations with the patient’s age. Additionally, we detected SBS17b, associated with fluorouracil chemotherapy treatment and damage inflicted by ROS, which could be due to the patient’s drug treatments. However, most SBSs do not yet have a known etiology. Further studies are needed to identify the etiology of these SBSs and increase the value of mutational signature analysis for personalized medicine. After filtering the variants, we identified five SNVs potentially associated with the development of cancer in our patient: *PIK3CA*, *TP53*, *NFKB2*, *ATM*, and *BTK*. Pathogenic mutations in *PIK3CA* and *TP53* were hypothesized to be drivers of the cancer. Because the sample’s tumor purity was higher than 80%, the mutation in *TP53* seemed to be present in all the cancer cells (allele frequency 55.71%), while the mutation in *PIK3CA* was present in a large percentage of them (allele frequency 34.17%). We also observed large chromosomal duplications and deletions in several chromosomes, highlighting the genomic instability of the tumor sample. These alterations presented a *log2* between -1 and 1, except in rare cases, which indicated their presence in one or more tumor cell subpopulations but not in the entire population of tumor cells. Given the large size of these alterations, we hypothesize that several genes and regulatory elements are involved and contribute to the cancer phenotype. To the best of our knowledge, this report is the first scientific paper showing chromosome alterations in apocrine breast cancer using short-read and long-read sequencing approaches.

Our RNA-seq analysis characterized the tumor sample’s gene expression profile. The results supported the immunohistochemical analysis in classifying the breast cancer subtype. Three different clustering approaches were used to achieve the goal: 1) Hierarchical clustering using Euclidean distance and complete-linkage method with *PGR*, *ESR1*, *HER2*, and *MKI67* genes, 2) *t-SNE* with PAM50 genes, 3) *t-SNE* with 18987 coding genes. All three methods clustered our sample with the HER2+ samples. This result, combined with the immunohistochemical analysis, led us to categorize this breast cancer sample as a HER2-positive subtype, although no *HER2* amplification at FISH was found. These promising results with clustering approaches highlight the need for a machine learning model running on gene expression data to improve the classification of breast cancer subtypes.

The results from the NGS and genomic/transcriptomic analysis confirmed our previous treatment choice but also opened the way for further treatments. Given the evidence of a strong intracellular driver (i.e. *PIK3CA*) combined with an inactivating *TP53* mutation, conventional chemotherapy is a more suitable candidate than anti-androgen or anti-HER2 therapy to stop neoplastic progression. However, if there is further progression, then the positivity of RNA for HER2 and AR pathways will support the use of anti-HER2 drug conjugates (25) or anti-androgenic treatments, possibly combined with off-label use of anti-PIK3CA treatment (26).

Although WGS and RNA-seq demand sophisticated infrastructure and expertise, they provide a comprehensive molecular characterization of the tumor. Our report and bioinformatics analysis offer an innovative personalized omics approach that could complement standard clinical practices and serve as a foundation for further fundamental research.

## Conclusion

This case study presents a complex picture of a 56-year-old woman patient with oligometastatic breast cancer with an intermediate phenotype between HER2-positive and triple-negative. The slow clinical course allowed the use of a sequential rather than all-in approach.

The genomic-bioinformatic analysis revealed five SNVs potentially associated with the development of cancer in the patient, as well as large chromosomal duplications and deletions. Somatic SNV mutations were associated with the patient’s age and chemotherapy treatment. RNA-seq analysis supported immunohistochemical analysis in classifying the breast cancer subtype as HER2-positive, although the disease was clinically defined as HER2-low. Overall, the study provides valuable insights into the complex genomic and molecular landscape of breast cancer with partial apocrine differentiation, and emphasizes the need for personalized and comprehensive approaches in cancer research and treatment.

## Data availability statement

The raw data supporting the conclusions of this article will be made available by the authors, without undue reservation.

## Ethics statement

The research was conducted in accordance with the principles embodied in the Declaration of Helsinki, in accordance with the Guidelines ICH-GCP and the applicable regulations. The study was approved by the Ethical Committee of the Regional Hospital “U. Parini”. The patients/participants provided their written informed consent to participate in this study. Written informed consent was obtained from the participant/patient(s) for the publication of this case report.

## Author contributions

ACa, BP, ACo, MS, AP, MV, SG conceptualized and designed the research. MV, MM, ST, VP, UF performed sample collection and sequencing. ACo, BP, FL, DC, FF carried out bioinformatic data analysis and tables/figures creation. ACo, BP, AP conducted manuscript drafting. ACa, MS, AP, MV, ST, UF, ACo, BP, PF, VA, AM, PC reviewed the written manuscript and participated in the modification. All authors contributed to the article and approved the submitted version.

## Supplementary material

Supplementary Figure 1 Case report timeline. Presented according to CARE guidelines. The dotted line is for ongoing treatments.
