# Supplementary material for: Multiomic analysis of HER2-enriched and AR-positive breast carcinoma with apocrine differentiation and an oligometastatic course: a case report
Source: Front Oncol. 2023 Jul 31;13:1240865. doi: 10.3389/fonc.2023.1240865 (PMC10424694; doi:10.3389/fonc.2023.1240865)
Supplement: Supplementary Figure 1 — Case report timeline. Presented according to CARE guidelines. The dotted line is for ongoing treatments. [file DataSheet_1.docx]

***Supplementary Material***

Multiomic analysis of HER2-enriched and AR-positive breast carcinoma with apocrine differentiation and an oligometastatic course: A case report.

**Brando Poggiali^1^, Agostino Ponzetti^3^, Marica Malerba^1^, Fabio Landuzzi^1^, Federica Furia^1^, Debora Charrance^1^, Sara Trova^2^, Vittoria Perseghin^1^, Patrizia Falcone^3^, Valentina Alliod^3^, Alessandra Malossi^3^, Pierpaolo Carassai^3^, Ubaldo Familiari^3^, Manuela Vecchi^2^, Stefano Gustincich^2^, Marina Schena^3^, Andrea Cavalli^1,4^*^†^ & Alessandro Coppe^1^*^†^**

^1^ Computational and Chemical Biology, Istituto Italiano di Tecnologia, CMP3VdA, Via Lavoratori-Vittime del Col du Mont, N. 28, 11100 Aosta, Italy

^2^ Non-Coding RNAs and RNA-Based Therapeutics, Istituto Italiano di Tecnologia, CMP3VdA, Via Lavoratori-Vittime del Col du Mont, N. 28, 11100 Aosta, Italy

^3^ Oncologia, Ematologia oncologica – Ospedale Umberto Parini, Viale Ginevra N. 3, 11100 Aosta, Italy

^4^ Centre Européen de Calcul Atomique et Moléculaire (CECAM), Ecole Polytechnique Fédérale de Lausanne, 1015 Lausanne, Switzerland

***Correspondence:** Andrea Cavalli, [andrea.cavalli@iit.it](mailto:andrea.cavalli@iit.it), Alessandro Coppe [alessandro.coppe@iit.it](mailto:alessandro.coppe@iit.it)

**^†^** These authors have contributed equally to this work and share last authorship.

#

# **Materials and Methods**

1. **Sample collection and DNA extraction**

Extraction of genomic DNA was performed from 200 µl of fresh whole blood using the MagCore® HF16 Automated Nucleic Acid Extraction Kit on the MagCore® HF16 Plus instrument (Diatech Labline) following manufacturer instructions.

After the excision of a metastatic lymph node, a fresh tumor samples was collected by the pathologist and immediately snap-frozen. Tumor cellularity was assessed on 5-µm-thick sections cut with Leica CM1950 cryotome, collected on a glass slide, and stained with a rapid Hematoxylin & Eosin staining. The tumor tissue sample exhibited a tumor cellularity >80% and did present necrosis. For genomic DNA extraction, 5 sections of 10 µm were cut and collected in a tube. Tumor sections were incubated with 400 µl of GT Lysis Buffer and 20 µl of Proteinase K (RBC Bioscience) at 55 °C for 1 hour for tissue lysis, then genomic DNA was automatically extracted with the MagCore® HF16 Plus instrument (Diatech Labline).

High Molecular Weight (HMW) gDNA extraction for long-reads sequencing was performed using 10 sections of 20 µm with the Monarch® HMW DNA Extraction Kit for Tissue (New England BioLabs), following the manufacturer’s instructions. Total RNA was extracted from 10 sections (15 µm thick) of the tumor sample following tissue homogenization with a mortar and pestle on dry-ice as well as a needle and syringe, using the RNeasy® Mini Kit (Qiagen, Venlo, Netherlands) according to the user manual.

Total RNA was extracted from 10 sections (15 µm thick) of the tumor sample following tissue homogenization with a mortar and pestle on dry-ice as well as a needle and syringe, using the RNeasy® Mini Kit (Qiagen, Venlo, Netherlands) according to the user manual.

1. **Nucleic Acid quality assessment and Illumina Sequencing**

Extracted gDNA and RNA were quantified using Nanodrop One^C^ spectrophotometer (Life Technologies, Thermo Fisher Scientific) to assess purity ratios and with Qubit Fluorometer 4.0 (ThermoFisher Scientific) to assess nucleic acid concentration (ng/µl) by using Qubit™ dsDNA High Sensitivity Assay kit for gDNA, Qubit™ DNA Broad Range Assay Kit for HMW gDNA, and Qubit™ RNA Broad Range Assay Kit for RNA (Invitrogen™, Thermo Fisher Scientific). The quality of gDNA, HMW gDNA and RNA were also assessed for fragmentation grade with the 4200 TapeStation system (Agilent Technologies), using the Genomic DNA ScreenTape and the RNA ScreenTape, respectively.

Whole Genome Sequencing of gDNA extracted from both blood and tissue samples was performed on an Illumina NovaSeq 6000 platform (Illumina Inc., CA, USA) to generate paired 151 base-pair reads. Library preparation and its sequencing were executed simultaneously for the gDNA extracted from the blood and the tissue samples using barcode adapters. Briefly, 350 ng of gDNA was used for library preparation with Illumina DNA PCR-Free Prep, Tagmentation library preparation kit (Cat number 20041795), and IDT® for Illumina® DNA/RNA Unique Dual Indexes Set B, Tagmentation (96 Indexes, Cat number 20027214). Library preparation was performed using a T100 Thermal Cycler instrument (Bio-Rad) and standard laboratory equipment. Libraries were pooled by mass in order to improve the balance of the coverage across samples within the same run.

Whole Transcriptome Sequencing was conducted on RNA extracted from tissue sample. RNA-seq libraries were conducted following DNase treatment and ribosomal transcript depletion of total RNA extracts, using the Illumina Total RNA Prep with Ribo-Zero Plus (Cat number 20040529), according to the manufacturer’s instructions. Whole transcriptome analysis was conducted on an Illumina NovaSeq 6000 platform (Illumina Inc., CA, USA) to generate paired 151 base-pair reads.

1. **Whole-genome sequencing (Illumina) data analysis (Figure S1).**

The demultiplexing step was achieved by using bcl2fastq (v2.20.0.422) and fastq files were generated. Then to align the germline and somatic paired-end reads to the reference genome (hg38), we utilized the NVIDIA Clara™ Parabricks® pipeline (3.8 version) with BWA-Mem [(1)](https://paperpile.com/c/GYfWoN/5C6O). Subsequently, we called germline and somatic variants using the NVIDIA Clara™ Parabricks® germline pipeline (3.8 version) with GATK HaplotypeCaller [(2)](https://paperpile.com/c/GYfWoN/KRLD) and the NVIDIA Clara™ Parabricks® somatic pipeline (3.8 version) with Mutect2 , respectively. The identified variants were annotated using SnpEff (5.0 version) [(1,3)](https://paperpile.com/c/GYfWoN/5C6O+8HzX), Annovar (databases updated to 23/11/2022)[(4)](https://paperpile.com/c/GYfWoN/KlaH), and Cosmic (Catalogue of Somatic Mutations In Cancer, CosmicCodingMuts.vcf.gz file, updated to 17/01/2022)[(5)](https://paperpile.com/c/GYfWoN/VhLj). To retain only the variants that play a significant role in cancer development and progression, we filtered the annotated variants through multiple steps. Specifically, we selected variants with "HIGH/MODERATE" impact according to SnpEff, a frequency <5% in GnomAD (3.0 version)[(6)](https://paperpile.com/c/GYfWoN/Cxvi), and a high-quality score ("PASS") based on FilterMutectCalls (gatk-4.1.0.0 version). Finally, we used the cancer_gene_census.csv file (updated to 15/12/2022) to report only the variants present in the Cancer Gene Census Project in the final vcf file.[(2)](https://paperpile.com/c/GYfWoN/KRLD)


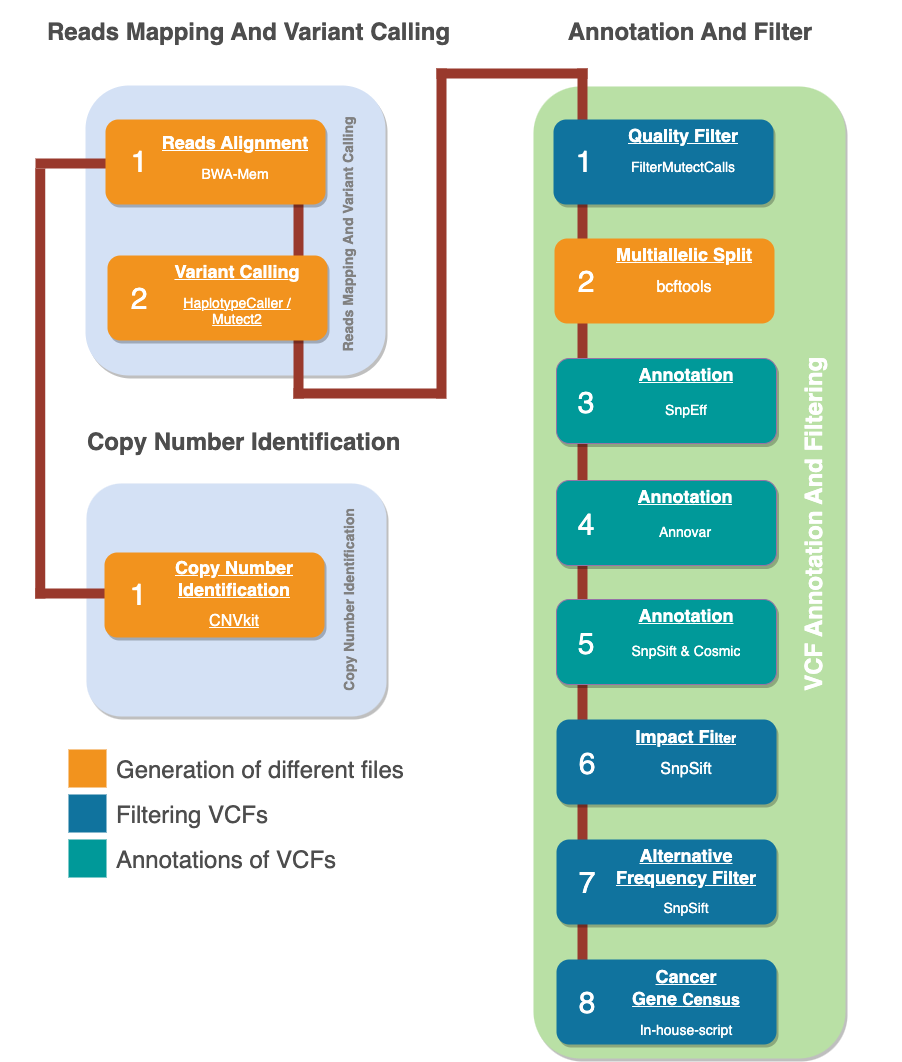


**Figure S1.** Whole-genome sequencing (Illumina) data analysis schema.

1. **Whole-genome sequencing (Oxford Nanopore Technologies) data analysis (Figure S2).**

HMW gDNA was used for Nanopore sequencing. The DNA Integrity Number (DIN) was assessed by the 4200 TapeStation system (Agilent Technologies) and was >7 (DIN = 9.5) as required for Nanopore sequencing. The HMW gDNA was further fragmented using a needle (G21) and syringe. Library preparation was performed using the DNA Ligation Sequencing Kit (SQK-LSK110 according to the manufacturer’s instructions. Long-reads sequencing was performed on the FLO-PRO002 flow cell (R9.4.1) using the PromethION 24 sequencer (Oxford Nanopore Technologies, ONT) following manufacturer’s instructions. A total of 300 ng of the library was obtained. Initially, 150 ng were directly loaded on the flow cell and after 36 hours of run a second loading with 150 ng was required. The sequencing experiment generated hundreds of raw fast5 sequencing files which were basecalled using Guppy basecaller (Version 6.2.1) with the super-accurate model “dna_r9.4.1_450bps_sup.cfg”. The resulting FASTQ files were merged into a single FASTQ file. Nanostat and Nanoplot were used to perform sequencing quality control and statistics [(7)](https://paperpile.com/c/GYfWoN/quGG). After that, reads were aligned to the reference genome GRCh38 using Minimap2 tool (version 2.17). The generated SAM file was converted into BAM format, which was sorted and indexed using samtools (Version 1.13) [(8)](https://paperpile.com/c/GYfWoN/QKYr). Genome coverage statistics were also generated by samtools.For tumor mutational burden (TMB) the TMB tool (https://github.com/bioinfo-pf-curie/TMB) implemented by Institut Curie was used. Only the non-synonymous mutations that have passed the quality filters and the effective genome size of the GRChg38 reference genome were considered.

For the Microsatellite Instability (MSI) analysis we have used the MSIsensor tool [(9)](https://paperpile.com/c/GYfWoN/KXKz) using paired tumor-normal sequencing data and considering a selected set of microsatellite sites.

1. **Copy Number Alteration analysis (Figure S1-S2).**

CNVkit [(10)](https://paperpile.com/c/GYfWoN/U1m5) software (Version 0.9.9) [(11)](https://paperpile.com/c/GYfWoN/xVtd) was used to detect Copy Number Variation and Alteration from blood and tumor BAM files obtained with Illumina sequencing and tumor BAM file obtained from Oxford Nanopore Technology. A coverage reference was built using 3 blood samples with the CNVkit command batch. Then CNV analysis was performed using a window of 750 bp and the aforementioned reference. In order to detect duplicated or deleted tumoral genes we filtered the chromosome region, present in the .cns file format obtained from CNVkit analysis, using a log2 upper threshold of 0.3 and a down threshold of -0.4. Only duplicated and deleted regions with a weight higher than 100 were kept. Then we filtered only for the genes present in the cancer_gene_census.csv file (updated to 15/12/2022).

For the CNA analysis performed on Nanopore data, CNVkit software was run using a window of 750 bp and a flat reference since no healthy sequenced genomes were available in our center when this analysis was performed.

Copy Number Variation plots were produced by the R package CNVmap (<https://github.com/BrandoPoggiali/CNVmap>).


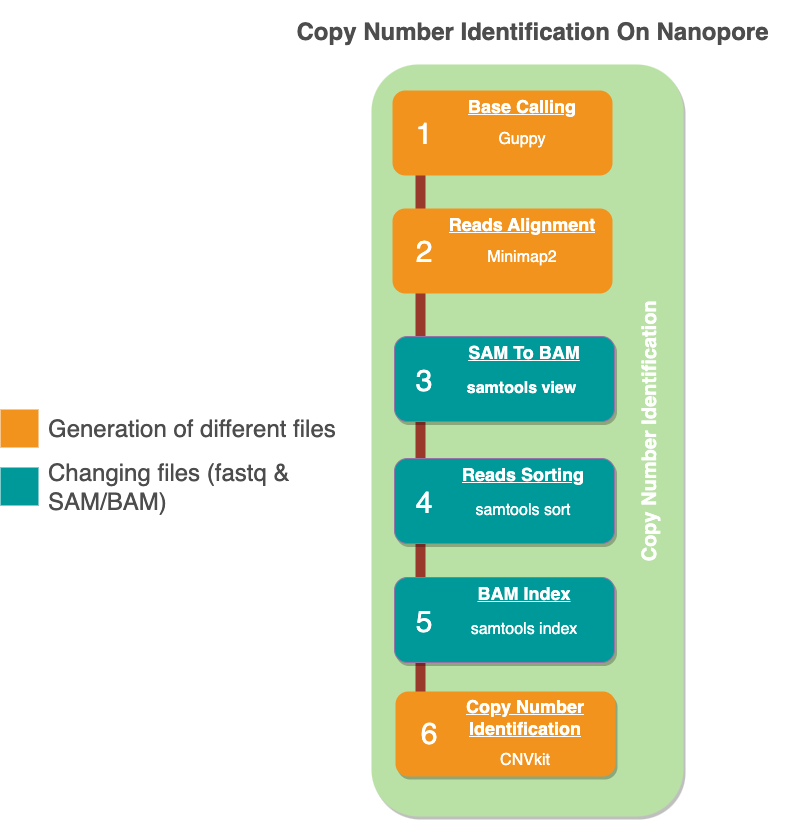


**Figure S2.** Schema of the Copy Number Identification workflow using Oxford Nanopore Technologies.

1. **Transcriptome data analysis (Figure S3).**

The RNA-Seq analysis began with the use of Trimmomatic [(12)](https://paperpile.com/c/GYfWoN/GtSv), a tool for trimming Illumina NGS data. The sliding window selected for this process was 4:20 and a minimum length of 25 nucleotides. Once the reads were trimmed, they were mapped to exons taken from version 106 of the GRCh38 Ensembl human transcriptome, which was downloaded from the Ensembl FTP server (https://ftp.ensembl.org/pub/release-106/gtf/homo_sapiens/). The software Hisat2 [(13)](https://paperpile.com/c/GYfWoN/myEa) was used for this mapping phase. After mapping, samtools view [(14)](https://paperpile.com/c/GYfWoN/ayzg) was utilized to convert the resulting SAM file to a compressed BAM file. Subsequently, the mapped reads were sorted based on their positions in the genome, and the final BAM file was indexed using the samtools index command. These sorting and indexing steps were necessary to facilitate the next phase, which involved counting the reads that aligned with different genes present in the human genome. The software HTSeq [(15)](https://paperpile.com/c/GYfWoN/L0gB) was used for this counting step. Finally, expression levels were estimated from the count data, and differential expression was tested using a model based on the negative binomial distribution with the DESeq2 R package [(15,16)](https://paperpile.com/c/GYfWoN/L0gB+3JAU).

Public data sets were downloaded to perform comparison and clustering with our sample:

- FASTQ files from 28 healthy breast tissue samples (of which 15 adjacent noncancerous tissues) downloaded from NCBI SRA (project accession numbers: PRJNA292118, PRJNA855324 and PRJNA839244).
- RNA-sequencing data from 1085 breast cancer patients obtained from the TCGA datasets (<https://xenabrowser.net/datapages/>), while the PAM50 subtype related to this group of patients were downloaded from the github repository: <https://github.com/yxchspring/GOEGCN_BRCA_Subtypes>.

FASTQ files of 28 healthy breast samples were analyzed as described above, then the reads count data were normalized together with reads count data obtained from our sample using DESeq2 R package.

Gene expression data of TCGA dataset were downloaded in HTSeq count data, which were converted into reads count data and normalized together with our sample.

Gene name conversion, heatmaps and t-SNE method were performed using “biomaRt”, “pheatmap” and “Rtsne” R-packages, respectively. The list of protein coding genes used for the t-SNE analysis was downloaded from HGNC database (<https://www.genenames.org/download/statistics-and-files/>).


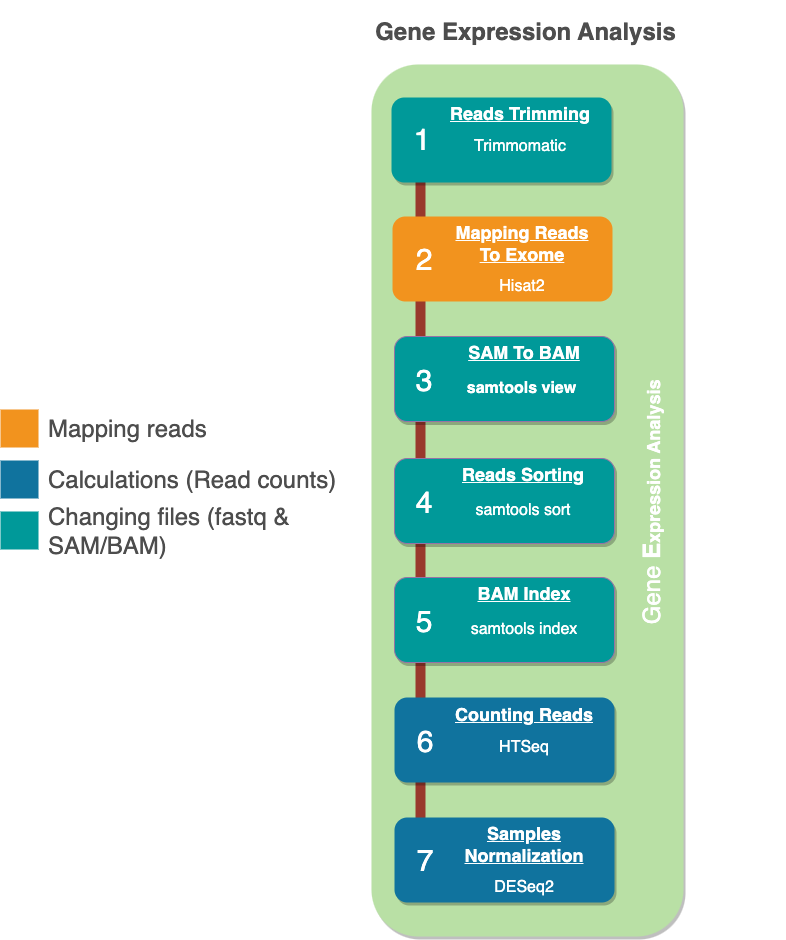


**Figure S3.** Gene expression analysis workflow.

**
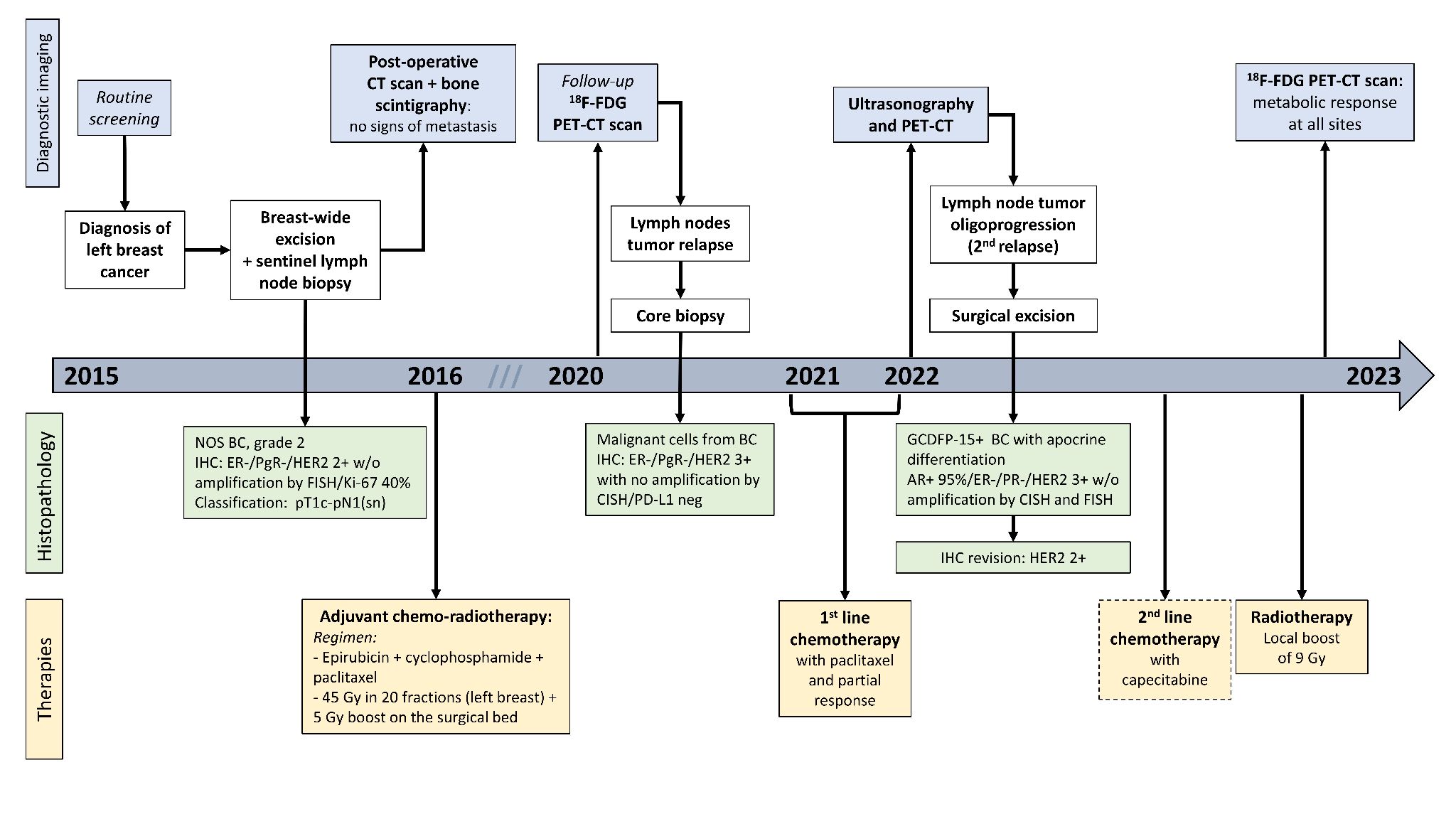
**

**Figure S4.** Case report timeline. Presented according to CARE guidelines. The dotted line is for ongoing treatments.

**References**

1. [Li H, Durbin R. Fast and accurate short read alignment with Burrows-Wheeler transform. Bioinformatics. 2009 Jul 15;25(14):1754–60.](http://paperpile.com/b/GYfWoN/5C6O)

2. [Van der Auwera GA, O’Connor BD. Genomics in the Cloud: Using Docker, GATK, and WDL in Terra. O’Reilly Media; 2020. 496 p.](http://paperpile.com/b/GYfWoN/KRLD)

3. [Cingolani P, Platts A, Wang LL, Coon M, Nguyen T, Wang L, et al. A program for annotating and predicting the effects of single nucleotide polymorphisms, SnpEff: SNPs in the genome of Drosophila melanogaster strain w1118; iso-2; iso-3. Fly . 2012 Apr-Jun;6(2):80–92.](http://paperpile.com/b/GYfWoN/8HzX)

4. [Wang K, Li M, Hakonarson H. ANNOVAR: functional annotation of genetic variants from high-throughput sequencing data. Nucleic Acids Res. 2010 Sep;38(16):e164.](http://paperpile.com/b/GYfWoN/KlaH)

5. [Bamford S, Dawson E, Forbes S, Clements J, Pettett R, Dogan A, et al. The COSMIC (Catalogue of Somatic Mutations in Cancer) database and website. Br J Cancer. 2004 Jul 19;91(2):355–8.](http://paperpile.com/b/GYfWoN/VhLj)

6. [Karczewski KJ, Weisburd B, Thomas B, Solomonson M, Ruderfer DM, Kavanagh D, et al. The ExAC browser: displaying reference data information from over 60 000 exomes. Nucleic Acids Res. 2017 Jan 4;45(D1):D840–5.](http://paperpile.com/b/GYfWoN/Cxvi)

7. [De Coster W, D’Hert S, Schultz DT, Cruts M, Van Broeckhoven C. NanoPack: visualizing and processing long-read sequencing data. Bioinformatics. 2018 Aug 1;34(15):2666–9.](http://paperpile.com/b/GYfWoN/quGG)

8. [Li H, Handsaker B, Wysoker A, Fennell T, Ruan J, Homer N, et al. The Sequence Alignment/Map format and SAMtools. Bioinformatics. 2009 Aug 15;25(16):2078–9.](http://paperpile.com/b/GYfWoN/QKYr)

9. [Jia P, Yang X, Guo L, Liu B, Lin J, Liang H, et al. MSIsensor-pro: Fast, Accurate, and Matched-normal-sample-free Detection of Microsatellite Instability. Genomics Proteomics Bioinformatics. 2020 Feb;18(1):65–71.](http://paperpile.com/b/GYfWoN/KXKz)

10. [Talevich E, Shain AH, Botton T, Bastian BC. CNVkit: Genome-Wide Copy Number Detection and Visualization from Targeted DNA Sequencing. PLoS Comput Biol. 2016 Apr;12(4):e1004873.](http://paperpile.com/b/GYfWoN/U1m5)

11. [van Riet J, Krol NMG, Atmodimedjo PN, Brosens E, van IJcken WFJ, Jansen MPHM, et al. SNPitty: An Intuitive Web Application for Interactive B-Allele Frequency and Copy Number Visualization of Next-Generation Sequencing Data. J Mol Diagn. 2018 Mar;20(2):166–76.](http://paperpile.com/b/GYfWoN/xVtd)

12. [Bolger AM, Lohse M, Usadel B. Trimmomatic: a flexible trimmer for Illumina sequence data. Bioinformatics. 2014 Aug 1;30(15):2114–20.](http://paperpile.com/b/GYfWoN/GtSv)

13. [Kim D, Langmead B, Salzberg SL. HISAT: a fast spliced aligner with low memory requirements. Nat Methods. 2015 Apr;12(4):357–60.](http://paperpile.com/b/GYfWoN/myEa)

14. [Danecek P, Bonfield JK, Liddle J, Marshall J, Ohan V, Pollard MO, et al. Twelve years of SAMtools and BCFtools. Gigascience [Internet]. 2021 Feb 16;10(2). Available from:](http://paperpile.com/b/GYfWoN/ayzg) <http://dx.doi.org/10.1093/gigascience/giab008>

15. [Anders S, Pyl PT, Huber W. HTSeq--a Python framework to work with high-throughput sequencing data. Bioinformatics. 2015 Jan 15;31(2):166–9.](http://paperpile.com/b/GYfWoN/L0gB)

16. [Love MI, Huber W, Anders S. Moderated estimation of fold change and dispersion for RNA-seq data with DESeq2. Genome Biol. 2014;15(12):550.](http://paperpile.com/b/GYfWoN/3JAU)
